# The first complete mitochondrial genome of macroparasite *Crassicauda magna* (Nematoda: Spirurida) from *Neophocoena sunameri* in ningbo, China

**DOI:** 10.1080/23802359.2021.1976688

**Published:** 2021-09-22

**Authors:** Ying Qiao, Xiaowan Ma, Shengping Zhong, Yongze Xing, Xuyang Chen, Bingyao Chen

**Affiliations:** a Key Laboratory of Tropical Marine Ecosystem and Bioresource, Fourth Institute of Oceanography, Ministry of Natural Resources, Beihai, China; b Institute of Marine Drugs, Guangxi University of Chinese Medicine, Nanning, China; c Jiangsu Key Laboratory for Biodiversity and Biotechnology, College of Life Sciences, Nanjing Normal University, Nanjing, China

**Keywords:** Mitochondrial genome, *Crassicauda magna*, *Neophocoena sunameri*

## Abstract

Members of the genus *Crassicauda* (Nematoda: Spirurida) are macroparasites infect the body tissues of whales and dolphins. However, limited information is available on morphological descriptions and phylogenetic studies of the worms. In present study, we report the first complete mitochondrial genome of *Crassicauda magna* from *Neophocoena sunameri* in Ningbo, Zhejiang Provence, China. The mitogenome has 13,605 base pairs (74.97% A + T content) and is made up of a total of 36 genes (12 protein-coding, 22 transfer RNAs, and 2 ribosomal RNAs). This study will provide useful molecular information for addressing taxonomic and evolutionary issues in *Crassicauda* sp..

*Crassicauda* spp. are the most unknown macroparasites which parasite the whales and dolphins. They often occur under subcutaneous tissues of the neck region, mammary glands, cranial sinuses and the urogenital system (Lambertsen 1986; Geraci and St. Aubin 1987; Jabbar et al. 2015; Johnston and Mawson 2021). For its large size and difficulties in the dissection of structurally complete samples from the tissues of hosts, lacking of data about morphological descriptions and molecular information limits the phylogenetic and taxonomic studies of the worms (Jabbar et al. 2015).

Specimens of *Crassicauda magna* isolate Ningbo were retrieved from three dead finless porpoise *Neophocoena sunameri* which stranded in Ningbo, ZheJiang Province, China. The specimen was deposited at Fourth institute of Oceanography, Ministry of Natural Resources, China as Parasite Sample No.: PSN-CmNb01 and was under the charge of Ying Qiao (qiaoying0618@hotmail.com). The total genomic DNA was extracted using an EasyPure^®^ Marine Animal Genomic DNA Kit (Transgen, China) following manufacturer’s instructions. DNA libraries (350 bp insert) were constructed with the TruSeq NanoTM kit (Illumina, San Diego, CA, USA) and were sequenced (2 × 150 bp paired-end) using HiSeq platform at BGI Company, China. Mitogenome assembly was performed by MITObim (Hahn et al. 2013).The complete mitogenome of *Anisakis pegreffii* GenBank accession number: NC_034329) was chosen as the initial reference sequence for MITObim assembly. Gene annotation was performed by MITOS (http://mitos2.bioinf.uni-leipzig.de).

The complete mitogenome of *Crassicauda magna* isolate Ningbo (GenBank accession number:MZ222136.1) is 13,605 bp in length and it contains 2 ribosomal RNA genes, 22 transfer RNA genes, a putative control region and 12 of the typical protein coding genes, lacking the Atpase subunit 8 (atp8) gene. A total of 36 genes were annotated and 227 nucleotides were putative control region. The overall base composition of the mitogenome is estimated to be A 22.43%, T 52.43%, C 7.23% and G 17.78%, with a high A + T content of 74.97%. The phylogenetic analysis constructed from homologous sequences of the 18 s ribosomal RNA gene revealed the parasite clustered with the *Crassicauda* species (Figure 1). This is the first sequenced mitogenome in *C. magna* from finless porpoise *N. sunameri* in China, which is useful for the better understanding the phylogenetic and taxonomic studies of *Crassicauda* sp.

**Figure 1.**
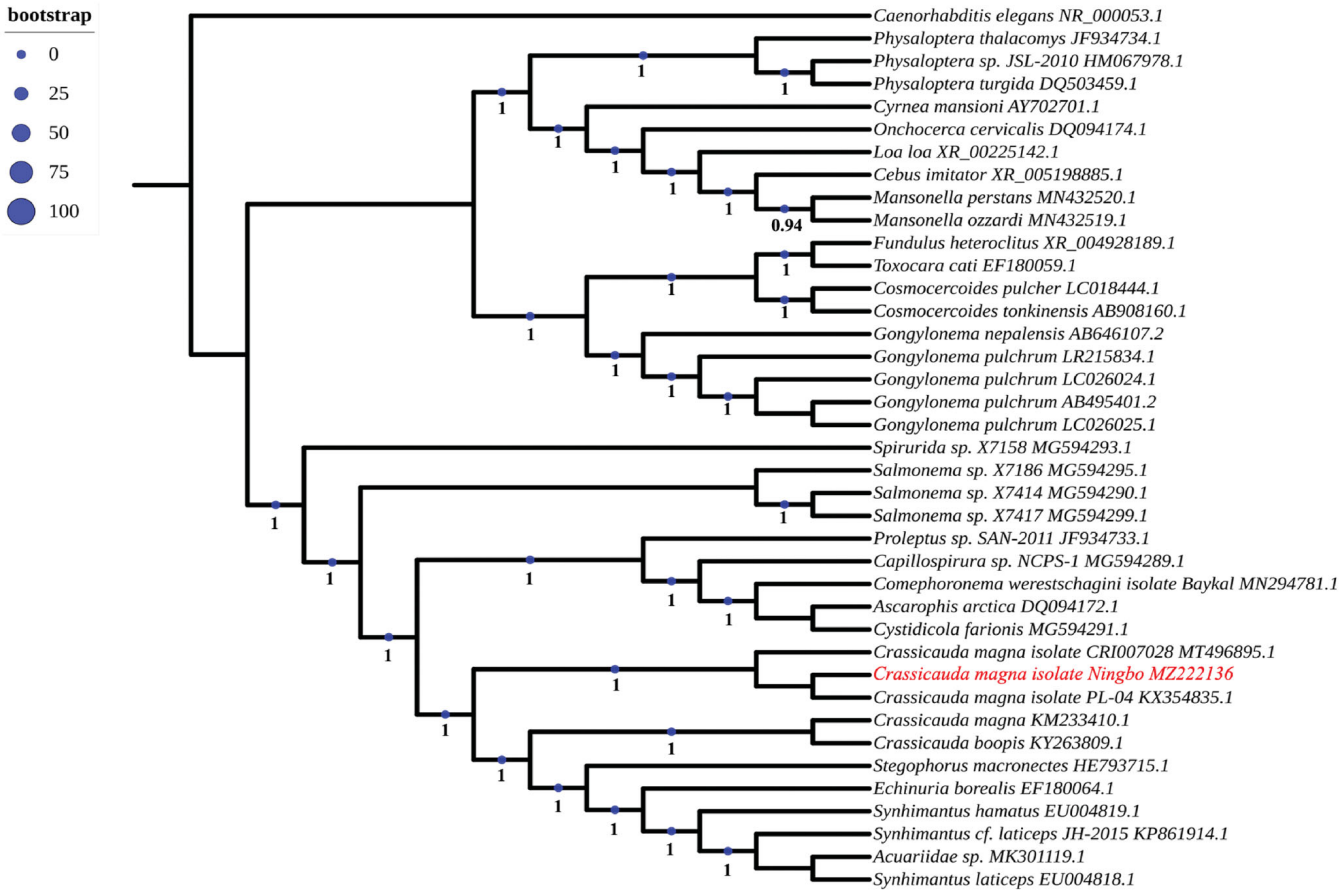
Phylogenetic tree of 18 s rRNA with homologous sequences. The18s rRNA sequences were downloaded from GenBank and the phylogenic tree was constructed by maximum-likelihood method in PhyML 3.0 with Smart Model Selection. The Akaike Information Criterion model and Subtree Pruning and Regrafting were used to improve the tree.

## Data Availability

The data that support the findings of this study are openly available in [National Center for Biotechnology Information] at [https://www.ncbi.nlm.nih.gov/nuccore], reference number [MZ222136.1]. The associated BioProject, SRA and BioSample numbers are PRJNA731245, SRR14706679, and SAMN19276108, respectively.
